# A systematic review of non-surgical management in Peyronieʼs disease

**DOI:** 10.1038/s41443-022-00633-w

**Published:** 2022-10-26

**Authors:** Sulaiman Hayat, Oliver Brunckhorst, Hussain M Alnajjar, Onur Omer Cakir, Asif Muneer, Kamran Ahmed

**Affiliations:** 1 MRC Centre for Transplantation, Guy’s Hospital Campus, King’s College London, King’s Health Partners, London, United Kingdom; 2 Department of Urology, University College London Hospitals NHS Foundation Trust, London, United Kingdom; 3 Division of Surgery and Interventional Science, University College London, London, United Kingdom; 4 NIHR Biomedical Research Centre, University College London Hospital, London, United Kingdom; 5 Department of Urology, Sheikh Khalifa Medical City, Abu Dhabi, United Arab Emirates; 6 Male Genital Cancer Centre, University College London, London, United Kingdom; 7 Khalifa University, Abu Dhabi, United Arab Emirates; 8 Department of Urology, King’s College Hospital NHS Foundation Trust, London, United Kingdom

**Keywords:** Sexual dysfunction, Combination drug therapy

## Abstract

The efficacy of many non-surgical treatments for Peyronie’s disease is unclear. This systematic review aims to critically assess the currently available options and provide a recommendation for treatment based on this. A systematic literature search utilising the Medline (Pubmed), Embase, global health and Cochrane library databases was conducted up to May 2021. All randomised controlled trials assessing non-surgical treatment modalities for Peyronie’s Disease were included. Individual study risk of bias was evaluated using the Cochrane tool and GRADE was used to assess evidence strength. Outcome measures were the change in penile curvature (degrees), plaque size (volume or size), International Index of Erectile Function score, pain scores and change in penile length. Prospero registration number: CRD42017064618. Amongst the 5549 articles identified, 41 studies (42 reports) were included. Seven different oral treatment options including vitamin E supplementation showed evidence for improving outcomes such as penile curvature and plaque size. Of the intralesional treatments, Collagenase Clostridium Histolyticum showed evidence for improving penile curvature (Range: 16.3–17 degrees, moderate level certainty of evidence). Intralesional Interferon demonstrated some improvement in curvature (Range: 12–13.5 degrees), plaque size (Range: 1.67–2.2 cm^2^) and pain, whilst intralesional calcium channel blockers such as Verapamil showed variable evidence for changes in the plaque size and pain. Extracorporeal Shockwave Therapy consistently demonstrated evidence for improving penile pain in stable disease, and two mechanical traction devices improved curvature. Iontophoresis, topical medications, and combination therapies did not demonstrate any consistent improvements in outcome measures. Intralesional options demonstrate the best potential. Overall, results varied with few high-quality randomised trials present.

## Introduction

Peyronie’s disease (PD) is an acquired connective tissue disorder characterised by fibrosis of the tunica albuginea resulting in the development of penile deformity, penile pain and penile shortening [1]. This can lead to not only sexual dysfunction, but also can be distressing for the patient, it can lead to relationship difficulties, increased anxiety over sexual performance and depression [2, 3]. This is compounded by a high estimated prevalence of between 3.2–11.8%, being [4, 5] even greater in certain subgroups such as those with diabetes mellitus (20%) [6] and post radical prostatectomy (16%) [7].

Whilst the exact pathophysiology of PD still remains unclear, it is believed microvascular trauma, initiated by penile damage during sexual activity or due to repetitive minor trauma, leading to increased proliferation of fibroblasts and recruitment of profibrotic mediators leads to an excessive deposition of collagen. [1]. This later remodels into a dense fibrotic plaque causing the onset of penile curvature [8, 9]. It is the disruption of this proposed pathway, that a number of the medical therapies have attempted to target.

However, the role of non-surgical treatments in both the acute and chronic setting remains unclear. The mainstay of treatment of PD remains focussed on the surgical correction of the penile deformity that develops once the patient is in the chronic phase. Yet, the desire to modify and influence the development of the plaque in the acute setting, preventing its development and effect on penile form and function is high. A wide range of options have been trialled to date, ranging from mechanical traction devices, intralesional injections, oral medication and atherotic treatment modalities. However, findings for the effectiveness of each remains varied with previous reviews focusing on individual modalities alone. Therefore, this systematic review aims to:

1. Identify current non-surgical treatment options for PD.
2. Assess the evidence for the effectiveness of the identified non-surgical therapy.
3. Provide recommendations for treatment based on the critical analysis of the current evidence base.

## Methods

This systematic review was performed following guidelines defined in the Preferred Reporting Items for Systematic Reviews and Meta-Analyses (PRISMA) statement [10, 11] and was prospectively registered, PROSPERO registration number: CRD42017064618. Utilising the PICO (population, intervention, control, and outcome) framework, the target population for this review was patients with acute and stable PD, the main interventions looked at were any non-surgical treatments, these were compared to control groups who had not received the relevant non-surgical treatment option and outcomes looked at included improvement in penile curvature, plaque size, pain, sexual function, and penile length. Only randomised controlled trials with more than 10 participants per cohort of intervention were included.

### Study eligibility criteria

All English randomised controlled trials analysing any non-surgical treatment option for PD in the acute and chronic phase were included. Conference abstracts were included if sufficient information was available in the abstract to conduct a thorough risk of bias evaluation of the article. The exclusion criteria consisted of review articles, all observational studies, non-randomised interventional studies and studies including less than 10 participants per cohort of intervention. Additionally, studies describing treatment options in congenital penile curvature, children under the age of 18 and animal studies were also excluded.

### Information sources and search

A systematic literature search utilising the Medline (Pubmed), Embase, global health and Cochrane library databases was conducted up to 26th May 2021 via a broad search strategy with no start date exclusion criteria. A broad search was conducted combining key words and MeSH terms for PD, management options and outcomes of interest (Supplementary Information: Appendix A). Subsequently, once individual treatment modalities were identified each was combined with the search strategy keywords, ensuring a comprehensive search of each. A reference review of articles and reviews was also subsequently conducted and grey literature was evaluated through conference abstracts searched via Embase.

### Study selection

Two reviewers (SH and OB) independently identified potentially relevant articles that arose from the search strategy once duplicates were removed, this process was managed using the online Covidence platform [12]. The full text of each potentially relevant article was subsequently obtained and reviewed against the inclusion criteria.

### Data collection and data items

Identified articles subsequently underwent data extraction by SH and OB onto a pre-defined and piloted extraction sheet. Study characteristics extracted included study design, intervention, number of patients in each arm of the study, treatment duration and follow up period. Outcome measures extracted for the effectiveness of intervention included the change in penile curvature in degrees, plaque size change measured in volume or size, sexual function via International Index of Erectile Function (IIEF) score [13], pain scores via visual analogue scores or subjective improvement of pain and penile dimensions such as stretched penile length or penile girth.

### Summary measures and synthesis of results

A meta-analysis was found to be unfeasible due to the heterogenous nature of study design, interventions and reporting measures and timeframes of outcomes. Therefore, a structured qualitative synthesis was conducted following the synthesis without meta-analysis (SWiM) guidelines [14]. Data synthesis was done on a per outcome basis, and due to the heterogeneity of data, vote counting was used to measure the direction of effect for each individual treatment modality identified, with study risk of bias rating and size of effect used to measure the clinical significance of findings.

### Study quality assessment

Individual study quality was evaluated using the Cochrane risk of bias tool [15]. Subsequently, the Grading of Recommendation, Assessment, Development, and Evaluation (GRADE) Working Group approach was used to evaluate the certainty of the evidence for each identified treatment modality on a per outcome basis (Supplementary Information: Appendix B) [16]. GRADE was not utilised for combination treatments identified as these varied greatly with few specific combinations evaluated beyond single studies.

## Results

### Study selection and result synthesis

**Fig. 1:**
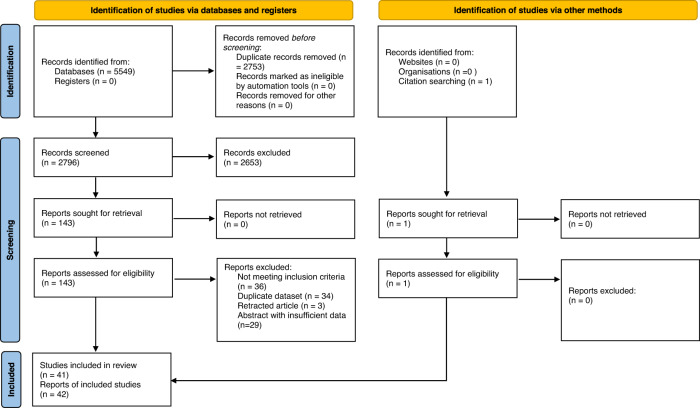
PRISMA Diagram. Flow diagram showing number of records identified, screened and including in review.

A total of 5549 articles were identified through the literature search with one additional article found following the reference review. Following duplicate removal and initial screening a total of 5406 articles were excluded. After full text review of the remaining 143 articles, 42 articles (41 studies) were included in the final review (Fig. 1). Results were classified into oral, intralesional, topical and combined treatments and further subdivided into specific treatment modality. Study characteristics are shown in Table 1 and GRADE evidence certainty profiles are included in Supplementary Information Appendix A with a summary in Table 2. The risk of bias evaluation for the individual studies is shown in Supplementary Information Appendix B.

**Table 1:** Study characteristics.

Study	Study design	Intervention/control	Acute or chronic	Duration of symptoms (months)	Follow up Period	Total number of patients	*N* intervention	*N* control
*Oral*								
Vitamin E								
Paulis et al. [20]	Non-blinded RCT	Peironimev-plus (vitamin E + other antioxidants…) + verapamil vs. verapamil	Acute?	12 to 13	6 months?	64	32	32
Paulis et al. [19]	Prospective RCT	Vitamin E + propolis + blueberry + verapamil + topical diclofenac vs without Vit E	Not defined	13.14 (intervention), 13.85 (control) (0.560)	?	70	35	35
Prieto Castro et al. [18]	Single blind RCT	Vit E (600 mg) + colchicine 1 mg vs. ibuprofen (control)	Acute	4.3 (intervention), 4.8 (control)	6 months	45	23	22
Safarinejad et al. [17]	Double blind RCT	4 groups, A- 300 mg Vit E BD, B - 1 gm carnitine BD, C- combination D - placebo	Chronic	18	16 months	236	A - 58 B - 59	C - 60 D - 59
Tamoxifen								
Teloken et al. [21]	Non-blinded RCT	Tamoxifen 20 mg Vs Placebo (3 month)	Not defined	?	4 months	25	13	12
*POTABA*								
Weidner et al. [23]	RCT, placebo controlled double blind	3 g POTABA vs. placebo	Acute	6	12 months	103	51	52
PDE5-i								
Ozturk et al. [25]	RCT	Vit E 400IU OD vs 50 mg sild OD	Acute	?	12–13 weeks	39	21	19
Colchicine								
Safarinejad [26]	Double blind RCT	colchicine 1 mg OD increased to 2.5 mg vs placebo	Not defined	14 (intervention), 16 (control)	16 months	84	42	42
Carnitine								
Safarinejad et al. REPEAT [17]	*Double blind RCT*	*4 groups, A- 300* *mg Vit E BD, B - 1* *gm carnitine BD, C- combination D - placebo*	*chronic*	*18*	*16 months*	*236*	*A - 58 B - 59*	*C - 60 D - 59*
Biagiotti and Cavallini [27]	Prospective RCT	Tamoxifen vs acetyl -l-carnitine	Mixed	?	3 months	48	24	24
Co-Enzyme Q10								
Safarinejad [28]	Double blind RCT	300 mg Co-Q10 vs placebo	Chronic	19.4	24 weeks	186	93	93
*Intralesional*								
Intralesional CCH								
Gelbard et al. [30]	RCT	9 injections. CCH vs. placebo injection and modelling vs. no modelling after injections	Chronic	?	18, 24, 36 weeks	147	111	36
Gelbard et al. (IMPRESS I and II) [29]	RCT double blind (combined analysis of IMPRESS I and II)	9 injections	Chronic	49.2	52 weeks	612	401	211
Interferon								
Hellstrom et al. [33]	Prospective single blind RCT	5 × 10^5 IFN for 6 injections	Chronic	24	at least 10 weeks	103	50	53
Kendirci et al. [34]	Prospective RCT	5 × 10^6 IFN for 6 injections	Not defined	?	12 weeks	39	19	20
Intralesional CCB								
Alizadeh et al. [40]	Prospective RCT	Verapamil vs pentoxy vs combination	Not defined	?	6 months	90	30	30
Sharma et al. [37]	RCT single blind	10 mg 6 injections vs placebo	Not defined	?	3 months	67	35	32
Shirazi et al. [39]	RCT	10 mg twice weekly for 12 weeks vs placebo	Not defined	20.6	24 weeks?	80	40	40
Soh et al. [38]	RCT single blind	10 mg Nicardipine vs NS control 6 bi-weekly injections	Not defined	?	48 weeks	74	37	37
*HA*								
Favilla et al. [42]	RCT double blind	intralesional injection of HA vs. injection of verapamil	Acute	?	12 weeks	132	63	69
Intralesional thiocolchicine								
Toscano et al. [43]	Prospective RCT - single blind	4 mg thiocolchicine vs. 5 mg verapamil intralesional	<18 months (chronic)	Stable for 5.48 months	1 month	25	13	12
*Topical*								
ESWT								
Chitale et al. [45]	RCT double blind	6 sessions	chronic	33	6 months	36	16	20
Hatzichristodoulou et al. [46]	RCT single blind	ESWT vs placebo (interposition of plastic preventing transmission)	chronic	?	4 weeks	102	51	51
Sokolakis et al. [48]	3-year outcomes for above study							
Palmieri et al. [47]	RCT double blind	four weekly sessions 12 weeks	Not defined	8.62 (placebo), 8.74 (intervention)	12, 24 weeks	100	50	50
Electromotive								
Di Stasi et al. [50]	RCT single blind	Electromotive with verapamil and dexa vs lidocaine	Not defined	?	1 month	73	37	36
Greenfield et al. [51]	Double Blind RCT	electromotive verapamil vs saline	chronic	19	3 months	42	23	19
Mehrsai et al. [52]	Prospective Randomised trial	electromotive of dexa and verapamil vs. intralesional	Not defined	?	1, 3 months	60	30	30
Montorsi et al. [49]	Prospective Randomised trial	electromotive dexa-orgotein and lidocaine	Not defined	8.5	3 months	40	*Cross-over study*	*Cross-over study*
Traction device								
Joseph et al. [54]	Non-blinded RCT	RestoreX traction device	Not defined	Stable for 32.85	3 months	90	29 (daily treatment), 23 (twice daily), 11 (thrice daily)	27
Moncada et al. [53]	Non-blinded RCT	Penimaster PRO traction device vs. no intervention	Chronic	19 (int), 20 (non-int)	3 months	80	41	39
Laser								
Allameh et al. [55]	Double blind RCT	Laser + verapamil vs. sham + verapamil	Mixed?	11.03 (control), 11.06 (int)	3, 9 months	38	18	20
Topical therapy								
Fitch III et al. [56]	RCT double blind	Topical verapamil	Not defined	?	9 months	57	N/A	N/A
Riedl et al. [57]	RCT double blind with crossover design at 8 weeks	Topical Liposomal Recombinant Human Superoxide Dismutase (2 mg)	Not defined	?	12 weeks	39	19	20
Twidwell and Levine [58]	RCT with crossover design	Topical gel h100 vs. placebo	Acute	7.2	3, 6 months	22	11	11
*Combination*								
Cavallini et al. [59]	Prospective Randomised + double blind trial	Verapamil + carnitine vs verapamil + tamoxifen	Chronic	13	6 months	60	30	30
Dell’Atti [61]	Prospective Randomised Trial	Verapamil (Group A) vs PDE5-I (Group B) vs combination (Group C)	Not defined	?	3 months	59	23	18 [17]
Favilla et al. [62]	Prospective Randomised trial	Verapamil vs. verapamil + antioxidant	Acute	3	3 months	105	52	53
Inal et al. [63]	Prospective RCT	5 × 106 interferon vs. Vit E vs both	Acute	10.6	6 months	30	10	10 [10]
Palmieri et al. [64]	Prospective double blind RCT	ESWT vs. ESWT + tadalafil	Acute	9	12 weeks, 24 weeks	100	50	50
Ralph et al. [65]	Randomised open label study	0.58 mg CCH 2 injections of 4 cycles + vacuum pump therapy vs same and modelling	chronic	?	36 weeks	30	15	15
Maretti and Canale [60]	Non-blinded RCT	TCARE + HEP + verapamil vs. TCARE	Mixed?	?	3 months	61	31	30
Cai et al. [66]	Prospective RCT	oral and intralesional HA vs. intralesional HA	Acute	?	3 months	81	41	40

Key for Table 1.

*RCT* Randomised control trial, *CCH* collagenase clostridium histolyticum, *CCB* calcium channel blocker, *ESWT* extracorporeal shockwave therapy, *PDEi-5* phosphodiesterase type 5 (PDE5) inhibitors, *HA* hyaluronic acid.

**Table 2:** GRADE summary.

Non-surgical treatment	Evidence for improvement? (GRADE level of evidence)				
	Penile curvature	Plaque size	Pain	IIEF	Penile length
Oral					
Vitamin E	Yes (Moderate)	Yes (Moderate)	No (Moderate)	Yes (Moderate)	N/A
Tamoxifen	No (Very Low)	No (Low)	No (Very Low)	N/A	N/A
POTABA	Yes (Low)	Yes (Low)	N (Very Low)	N/A	N/A
PDEi-5	No (Low)	No (Very Low)	Yes (Low)	Yes (Low)	N/A
Colchicine	No (Low)	No (Very Low)	No (Very Low)	N/A	N/A
Carnitine	Yes (Very Low)	Yes (Very Low)	No (Very Low)	No (Low)	N/A
Co-Enzyme Q10	Yes (Low)	Yes (Low)	No (Low)	Yes (Low)	N/A
Intralesional					
Intralesional CCH	Yes (Moderate)	N/A	No (Moderate)	Yes (Low)	Yes (Low)
Interferon	Yes (Very Low)	Yes (Low)	Yes (Low)	No (Low)	N/A
Intralesional CCB	No (Low)	Yes (Low)	Yes (Low)	Yes (Low)	N/A
HA	Yes (Low)	No (Very Low)	N/A	No (Very Low)	N/A
Intralesional thiocolchicine	Yes (Low)	No (Low)	N/A	No (Low)	N/A
Topical					
ESWT	Yes (Low)	Yes (Moderate)	Yes (Low)	Yes (Moderate)	No (Low)
Electromotive	Yes (Moderate)	Yes (Moderate)	Yes (Moderate)	No (Moderate)	No (Moderate)
Traction device	Yes (Moderate)	N/A	No (Moderate)	No (Moderate)	Yes (Moderate)
Laser	Yes (Moderate)	No (Moderate)	Yes (Moderate)	Yes (Moderate)	No (Moderate)
Topical therapy	No (Low)	Yes (Low)	Yes (Low)	N/A	Yes (Low)

Key for Table 2.

*CCH* collagenase clostridium histolyticum, *CCB* calcium channel blocker, *ESWT* extracorporeal shockwave therapy, *PDEi-5* phosphodiesterase type 5 (PDE5) inhibitors, *HA* hyaluronic acid.

### Oral therapies

#### Vitamin E

Vitamin E is a natural antioxidant which is believed to reduce collagen deposition and improve endothelial function [17]. Four randomised trials studies were identified assessing its effectiveness [17–20]. Plaque size was seen to be reduced in three of the studies (moderate quality of evidence) [18–20], however, the largest of the trials [17], with 236 patients failed to show any significant differences between the cohorts. When assessing penile curvature three randomised trials [18–20] identified benefit with two trials [19, 20] reporting numerical data ranging from improvements of 8.7 to 12.25 degrees (moderate quality of evidence). When assessing IIEF scores, only two studies [19, 20] identified any benefit (IIEF improvement of between 4.9 and 5.07). No studies assessed the impact of vitamin E on penile dimensions and no studies found any benefit for pain.

#### Tamoxifen

Tamoxifen is a nonsteroidal anti-oestrogen, believed to modulate transforming growth factor (TGF)β1 by fibroblasts and thereby affecting deposition of scar tissue [21]. One RCT assessed its effectiveness [21]. No significant improvements were seen in the penile curvature, pain, plaque size or IIEF scores with a low to very low quality of evidence. The penile dimensions were not assessed.

#### Potassium Paraaminobenozate (POTABA)

POTABA is an antifibrotic agent, believed to increase oxygen uptake by tissues, monoamine oxidase activity and glycosaminoglycans secretion [22]. One placebo controlled RCT assessing POTABA in 103 patients was found [23]. When assessing curvature improvement, subjective improvements were noted with POTABA (74.3% experienced improvement vs. 50%, *p* = 0.016). Additionally, plaque size was seen to be significantly reduced but no absolute figures were given. No significant differences in pain or IIEF scores were seen, and penile dimensions were not assessed.

#### Phosphodiesterase type 5 inhibitors (PDE5-I)

PDE5-I have been shown to reduce collagen/smooth muscle ratios and increase apoptosis in rat models [24]. A small RCT of 39 patients demonstrated improvements in IIEF scores (post-treatment IIEF scores 13.9 vs. 10.7, *p* = 0.028, increase in 3.8 vs. 0.89) [25]. Further small improvements in Visual Analogue Scores (VAS) for pain were seen. Penile dimensions were not recorded and no significant change in penile curvature or plaque size was noted.

#### Colchicine

Colchicine is believed to activate collagenase and decrease collagen synthesis, thereby having a potential role in PD [18]. A single placebo controlled RCT demonstrated no improvements in pain, curvature or plaque size [26]. No IIEF scores or penile dimensions were recorded.

#### Acetyl-L-carnitine

Oral acetyl-L-carnitine increases mitochondrial respiration and metabolism of fatty acid and free radicals, therefore a potential therapy for any oxidative disease [27]. One small RCT of 48 patients identified improvements in curvature (mean improvement 7.5° vs. 0.5°, *p* < 0.01) and plaque size (61 mm^2^ vs. 89.6 mm^2^) [27]. This was not reproduced by a larger trial of 236 men [17], with no evidence for improvements in pain or IIEF scores in either trial and neither study assessed penile dimensions.

#### Co-enzyme Q_10_

This lipid-soluble antioxidant has a potential effect in PD via inhibition of TGF-β1 production, thereby reducing scar formation. A single RCT of 186 patients identified some subjective improvements in curvature (60.5% vs. 17.1% *p* < 0.01), plaque size and IIEF scores (17.8 vs. 8.8 post treatment, *p* = 0.001), with no effect on pain [28]. Penile length was not assessed.

### Intralesional therapies

#### Collagenase clostridium histolyticum (CCH)

CCH is a purified bacterial enzyme which selectively breaks down collagen and therefore can break down or soften the plaque in PD [29]. It has generated a global interest as a non-surgical option, with two randomised trials identified conducted in patients with stable disease [29, 30]. Penile curvature improved objectively in both trials, including the IMPRESS I and II trials [29], which are placebo-controlled trials with a total of 612 patients (curvature improvement of 16.3° vs. 5.4°, *p* < 0.001, moderate quality of evidence). Sub-analysis of the data revealed that angle change was irrespective of the initial curvature (all above 30°), but a greater difference was seen in those with a disease duration of over two years [31]. An improvement of 16.3° in curvature was seen in the other RCT demonstrating consistent evidence for treating curvature [30]. Furthermore, the IMPRESS trials identified small, but statistically significant improvements in erectile function via their IIEF scores (improvement of 1.0, *p* < 0.05, low quality of evidence) [29]. Finally, small but statistically significant improvements in stretched penile length were seen in the IMPRESS trials (improvement of 0.4 cm) [29]. There is, however, no evidence of improvement of pain or plaque size in any of the studies.

#### Interferon-alpha 2B

Interferon decreases fibroblast production of collagen and increases collagenase production [32]. Two randomised trials were identified assessing its effectiveness [33, 34]. These studies have largely assessed patients with a curvature of greater than 40° with a low level of evidence. When assessing curvature improvement, a RCT of 103 patients demonstrated significant improvements (13.5° vs. 4.5°, *p* < 0.01) but with a very low level of evidence [33]. Plaque size was improved in both RCTs (reduction of 2.2 cm^2^ vs. 0.9 cm^2^, *p* < 0.001 and reduction of 1.67 cm^2^ vs. 0.73, *p* < 0.05). Pain was seen to be significantly improved in both randomised trials. However, the level of evidence for plaque size and pain was low. None of the studies assessed the effects on penile dimensions. However, there is a large variation in how measurements were conducted for plaque size, thereby providing a low evidence base for utilisation clinically. There is little evidence to support the use of interferon for improving erectile function and none for penile length.

#### Calcium channel blockers

Verapamil inhibits extracellular matrix molecules such as fibronectin and collagen and increases collagenase activity, thereby affecting plaque formation [35, 36]. Four randomised studies were seen utilising intralesional verapamil and nicardipine [37–40]. Penile curvature was not seen to be improved in any of the trials. When assessing plaque size improvement one study using nicardipine demonstrated significant objective improvements (12 vs. 0 mm, *p* < 0.01), pain and IIEF scores were also significantly affected by the treatment in this study [38]. No studies identified any benefit of verapamil in erectile function, pain, or penile dimensions.

#### Hyaluronic acid (HA)

HA is in the tunica albuginea and has effects on nutrient distribution within the tissue counteracting inflammatory cytokine activity [41]. One randomised trial of 132 patients identified demonstrated significant improvements of 4.6 degrees as compared to verapamil (*p* < 0.01) [42]. Plaque size and IIEF scores were not significantly improved. No penile dimensions or pain scores were recorded.

#### Thiocolchicine

The anti-inflammatory properties of colchicine are believed to be increased when injected directly into a penile plaque. A single small randomised trial of 25 patients demonstrated improvements in curvature (10.5° vs. 7.8°, *p* = 0.012) and plaque size, however little benefit was seen when assessing IIEF scores with pain or penile length not being assessed [43].

### Topical therapies

#### Extracorporeal shockwave therapy (ESWT)

ESWT is advocated to mechanically damage and remodel the plaque, as well as increase vascularity and inflammation locally, resulting in lysis and resorption of the plaque [44]. This has been extensively reported with three randomised studies (Table 2) [45–47] and one trial [46] also had a three year follow up study [48]. Curvature improved in one randomised study [47] (1.43°vs. increase of 1.8, *p* < 0.05), however the remaining two randomised trials failed to reproduce this with no statistically significant results and a low level of evidence. Plaque and IIEF score improvement was seen in only one randomised study [47], both outcome measures had a moderate level of evidence. When assessing pain two randomised trials demonstrated significant [46, 47] improvements in pain VAS ranging between 1–5.1 with a low level of evidence. None of the studies demonstrated improvement in penile length.

#### Transdermal electromotive administration of medication

Iontophoresis has been used to improve local absorption of topical medication with four studies identified assessing its use in PD [49–52]. However, these assessed different combinations of medications with two assessing the combination of dexamethasone and verapamil [50, 52], one verapamil alone [51] and one dexamethasone alone [49]. Combination treatment demonstrated objective curvature improvement in one randomised study (22° vs. 0°, *p* < 0.001 when compared against lidocaine) [50]. Plaque size was improved objectively in the same randomised trial (347 mm^2^ vs. 766 mm^2^, *p* = 0.001 when compared to lidocaine) [50]. Importantly, two other randomised trials saw no differences in curvature and plaque size [51, 52]. Pain was improved in two randomised trials [50, 52]. No improvements in IIEF score occurred in the one study assessing it [52]. Individual administration of medications showed improvements in curvature, plaque size and pain with dexamethasone [49].

#### Traction devices

Various types of traction devices have been utilised in PD to mechanically correct the penile deformity, with two RCTs found [53, 54]. The two studies assessed different devices, including a vacuum erection device (VED) [53] and an external traction device [54]. Both studies identified a benefit with respect to curvature. Neither of the studies assessed the effect on plaque size for the traction devices. One of studies demonstrated no benefit with respect to penile pain and IIEF scores but did identify improvement in stretched penile length (+1.5 cm vs 0 cm, *p* < 0.001) but not with respect to penile girth [54]. This is compared to the other study which did not look at pain or IIEF score, penile length and girth were not significantly affected [53].

#### Laser

Lower-intensity laser can reduce the levels of abnormal collagen in the scar tissue of PD. One double-blind randomised study was found using laser treatment for PD, comparing verapamil and sham treatment to verapamil and laser treatment [55]. There was a significant improvement of penile curvature at 12 weeks but results at 36 weeks were not significant. There were no significant improvements in plaque size. Reduction in pain was significant (VAS reduced by 2.7 vs. 1.1, *p* = 0.033). There was a significant improvement of IIEF scores (improvement of 7.1 vs. 1.3, *p* = 0.003). Penile length not measured.

#### Topical medications

Various topical applications of medications have been described with three randomised studies identified [56–58]. All assessed different topical medications including verapamil [56], liposomal recombinant human superoxide dismutase [57] and gel h100 [58]. Only topical verapamil demonstrated improvement in subjective curvature [56]. All three studies improved pain via non-validated questionnaires [56–58]. No studies demonstrated benefit with regards to plaque size or IIEF scores, but one identified small improvements in stretched penile length with gel h100 [58].

### Combination therapies

A total of 8 randomised studies were identified exclusively assessing the effectiveness of combination therapies, often utilising a mixture of oral antioxidants, intralesional and topical treatment modalities [59–66]. Every study assessed differing combinations of these, and whilst many studies [59–62] utilised intralesional verapamil no combination of treatments was assessed in more than a single study. There is therefore no consistent evidence for any combination utilised for penile curvature, plaque improvement, pain, IIEF scores or penile measurements.

## Discussion

Many systematic reviews looking at the medical treatment options for PD have been conducted in the past [67, 68]; however, this review is different in that it includes a broader range of the available medical treatment options and only includes randomised control trials, a meta-analysis was not conducted due to the heterogeneity of the data.

Surgical interventions are considered the gold standard treatment for PD, especially for the correction of penile deformity [69, 70]. In many studies looking at the efficacy of surgical treatment options for PD there is a lack of validated patient-reported outcomes looking at things such as the psychological impact of treatment, this partly explains why the American Urological Association guidelines for the treatment of PD rate the evidence basis for surgical treatment poorly [1]. However patient morbidity associated with surgery has led to increasing interest in medical interventions for PD, for example in cases of recurrent or residual curvature [71].

This systematic review presents the current randomised trial evidence base for all investigated non-surgical treatment modalities in PD. Critically evaluating only high quality randomised controlled trials it is still currently not possible to make strong clinical recommendations for the use of any modality, with little certainty in the current evidence base. However, despite this some non-surgical treatments have demonstrated potential in the current literature.

All oral therapies identified provided no consistent evidence with respect to any of the outcome measures evaluated, meaning they are cannot at present be recommended for clinical use. Intralesional CCH currently offers the only Food and Drug (FDA) approved medication for stable PD [72]. Both studies investigating this treatment showed significant improvements in penile curvature in the chronic phase and in those with curvature of greater than 30° meaning its clinical utilisation should be focused for this use. There was no significant benefit demonstrated when assessing plaque size, pain or penile length. This is in keeping with another recent review which included observational studies as well [73]. Interestingly many observational “real world” post-approval studies [74–76] have shown intralesional CCH to be of benefit in acute phases of PD, but these findings are yet to be confirmed in RCTs. Intralesional interferon, verapamil and hyaluronic acid have a limited evidence base and are currently cannot be recommended for treatment.

Several external therapies were identified with ESWT the most investigated treatment modality with three randomised trials mostly in the chronic phase. Interestingly, whilst not expected to be a predominant feature in the chronic phase of PD there appears to be an improvement in penile pain across two of the studies but with a low level of evidence. There is no reproducible evidence for other outcome measures evaluating and therefore the level of evidence for the use of ESWT, outside of treating pain, is limited. However, in a meta-analysis which included case-control studies and cohort studies as well as RCTs, plaque size and pain but not curvature were significantly improved using ESWT [77]. Interestingly two observational studies have demonstrated that ESWT can potentially improve penile curvature [78, 79]. Traction therapy has demonstrated improvements in penile curvature in two studies with different devices respectively [53, 54]. Iontophoresis demonstrated some improvements for penile curvature and plaque size [50] however this evidence is not consistent amongst the larger RCTs with differing medications used [51, 52]. Similarly, topical applications of medications present no consistent evidence for all outcome measures, with only small studies assessing each treatment option [56–58]. Finally, whilst various randomised trials have assessed combination therapies, no two trials have investigated the same combination of therapy, meaning little meaningful recommendation can be made for any individual combination treatment.

Although there is enormous interest in non-surgical treatment options of PD, there is still a paucity of data from large, well conducted randomised trials within the literature. Other reviews [73, 80, 81] of the literature have focused only on selected non-surgical treatments or have included studies other than randomised trials resulting in a varied and inconclusive evidence based which has at times conflicted with our findings. This systematic review provides the most up-to date and extensive summary of all non-surgical treatments for PD, including only high-quality randomised trials. This has resulted in a critical analysis of a large amount of evidence prior to making recommendations for treatment.

However, this review does have some limitations. Like any systematic review some references of value may have been missed during the search process. Furthermore, despite some randomised trials being conducted in various modalities, after assessment due to large heterogeneity, inconsistencies and bias a statistical synthesis of the results, via a meta-analysis was not possible. This additionally highlights that due to our wide inclusion criteria most studies identified had large differences with respects to both methodology and results obtained.

This systematic review has highlighted the current evidence base which has demonstrated some promising treatment options, this can guide what specifically needs to be done in terms of future research. Future research trials are needed to look at the currently available treatment modalities in more depth as well as new ones. Trials need to include a wider range of patients, for example more work is needed in looking at treatment in both acute and chronic PD. Whilst the desire for non-surgical treatments is within the acute setting, whereby you can alter the inflammatory process, many studies in the literature, particularly for intralesional and topical therapies have assessed patients in the stable phase of the disease [29, 30, 56–58]. This is therefore certainly one of the cohorts of patients that requires further investigation. Wide inclusion criteria in numerous studies have limited the applicability of any positive findings to specific patient groups. At present it is difficult to make any clinical recommendations for treatments specifically for acute and chronic phase of the disease. Different studies in this review have included patients with varying lengths of disease timeframe, therefore specific phases of the disease need to be looked at in studies with more restrictive inclusion criteria. Clinically the acute and chronic phases of PD are separate entities and have been seen to respond differently to similar treatments.

It is important that future research focuses on assessing the newer treatment modalities which have demonstrated potential such as HA. Also, more work is needed for treatments where the results are varied and conflicting, for example some studies have demonstrated that ESWT can treat penile curvature whereas some have showed no efficacy. However, it is also important clinically to identify cohorts of patients which would benefit from specific treatment modalities. This can be achieved by wide inclusion criteria with subsequent sub-group analysis to identify which patient demographics can potentially benefit from treatment.

## Conclusions

PD remains common and can a have significant impact on those affected. Despite numerous randomised studies investigating non-surgical options, there remains little evidence to support the widespread clinical use of any individual non-surgical treatment modality for many outcomes. However, some modalities appear to show potential, with ESWT and traction therapies demonstrating some improvements in improving pain and curvature respectively. Furthermore, intralesional therapies appear to currently demonstrate the best non-surgical treatment options available. There is some evidence for the use of CCH in stable disease with curvature over 30° and with HA offering a potential role in active disease but this is subject to further research. It is however clear that whilst many non-surgical treatment modalities have emerged, there is still a lack of good quality, randomised data for the majority of these, requiring more investigation to identify the best modality and patient cohort for utilisation.

## Supplementary information

Supplementary information
