# Supplementary material for: A systematic review of non-surgical management in Peyronieʼs disease
Source: Int J Impot Res. 2022 Oct 26;35(6):523–32. doi: 10.1038/s41443-022-00633-w (PMC10499596; doi:10.1038/s41443-022-00633-w)
Supplement: Supplementary file 1 — Supplementary information [file 41443_2022_633_MOESM1_ESM.docx]

**APPENDIX**

Appendix A – Full Search Strategy and Initial Identification Results

Search Results (up to 26^th^ May 2021)

Pubmed, Ovid (embase & global health) and Cochrane library

Broad:

(‘peyronie`s disease’ OR peyronie`s) AND (therapy OR intervention OR management OR treatment) AND (‘curvature’ OR ‘pain’ OR plaque OR ‘penile length’ OR 'Sexual function' OR 'Erectile Dysfunction' OR 'sexual dysfunction' OR ED )-1603 results, 2169 results, 8 results

Specific

**Vitamin E**

(‘peyronie`s disease’ OR peyronie`s) AND (‘vitamin E’) AND (‘curvature’ OR ‘pain’ OR plaque OR ‘penile length’ OR 'Sexual function' OR 'Erectile Dysfunction' OR 'sexual dysfunction' OR ED )-56 results, 68 results, 0 results

**Propionyl l carnitine**

(‘peyronie`s disease’ OR peyronie`s) AND (‘Propionyl l carnitine’) AND (‘curvature’ OR ‘pain’ OR plaque OR ‘penile length’ OR 'Sexual function' OR 'Erectile Dysfunction' OR 'sexual dysfunction' OR ED )-3 results, 8 results, 0 results

**Colchicine**

(‘peyronie`s disease’ OR peyronie`s) AND (‘**Colchicine**’) AND (‘curvature’ OR ‘pain’ OR plaque OR ‘penile length’ OR 'Sexual function' OR 'Erectile Dysfunction' OR 'sexual dysfunction' OR ED )-35 results, 127 results, 0 results

**POTABA**

(‘peyronie`s disease’ OR peyronie`s) AND (‘**POTABA**’) AND (‘curvature’ OR ‘pain’ OR plaque OR ‘penile length’ OR 'Sexual function' OR 'Erectile Dysfunction' OR 'sexual dysfunction' OR ED )-25 results, 48 results , 0 results

**Verapamil**

(‘peyronie`s disease’ OR peyronie`s) AND (‘**verapamil**’) AND (‘curvature’ OR ‘pain’ OR plaque OR ‘penile length’ OR 'Sexual function' OR 'Erectile Dysfunction' OR 'sexual dysfunction' OR ED )-109 results, 261 results, 1 result

**Liposomal recombinant**

(‘peyronie`s disease’ OR peyronie`s) AND (‘**liposomal recombinant**’) AND (‘curvature’ OR ‘pain’ OR plaque OR ‘penile length’ OR 'Sexual function' OR 'Erectile Dysfunction' OR 'sexual dysfunction' OR ED )-2 results, 4 results, 0 results

**Interferon**

(‘peyronie`s disease’ OR peyronie`s) AND (‘**interferon**’) AND (‘curvature’ OR ‘pain’ OR plaque OR ‘penile length’ OR 'Sexual function' OR 'Erectile Dysfunction' OR 'sexual dysfunction' OR ED )-65 results, 180 results , 0 results

**Collagenase Clostridium Histolyticum**

(‘peyronie`s disease’ OR peyronie`s) AND (‘**collagenase clostridium histolyticum**’) AND (‘curvature’ OR ‘pain’ OR plaque OR ‘penile length’ OR 'Sexual function' OR 'Erectile Dysfunction' OR 'sexual dysfunction' OR ED )-132 results, 296 results, 4 results

**Radiation**

(‘peyronie`s disease’ OR peyronie`s) AND (‘**radiation**’) AND (‘curvature’ OR ‘pain’ OR plaque OR ‘penile length’ OR 'Sexual function' OR 'Erectile Dysfunction' OR 'sexual dysfunction' OR ED )-55 results, 60 results, 0 results

**Extracorporeal shock wave therapy**

(‘peyronie`s disease’ OR peyronie`s) AND (‘**extracorporeal shockwave therapy**’) AND (‘curvature’ OR ‘pain’ OR plaque OR ‘penile length’ OR 'Sexual function' OR 'Erectile Dysfunction' OR 'sexual dysfunction' OR ED )-35 results , 30 results, 1 result

**Tamoxifen**

(‘peyronie`s disease’ OR peyronie`s) AND (‘**tamoxifen**’) AND (‘curvature’ OR ‘pain’ OR plaque OR ‘penile length’ OR 'Sexual function' OR 'Erectile Dysfunction' OR 'sexual dysfunction' OR ED )-19 results, 107 results, 0 results

**Procarbazine**

(‘peyronie`s disease’ OR peyronie`s) AND (‘**procarbazine**’) AND (‘curvature’ OR ‘pain’ OR plaque OR ‘penile length’ OR 'Sexual function' OR 'Erectile Dysfunction' OR 'sexual dysfunction' OR ED )-1 result, 17 results, 0 results

**Omega 3**

(‘peyronie`s disease’ OR peyronie`s) AND (‘**omega 3**’) AND (‘curvature’ OR ‘pain’ OR plaque OR ‘penile length’ OR 'Sexual function' OR 'Erectile Dysfunction' OR 'sexual dysfunction' OR ED )-NO results, 9 results , 0 results


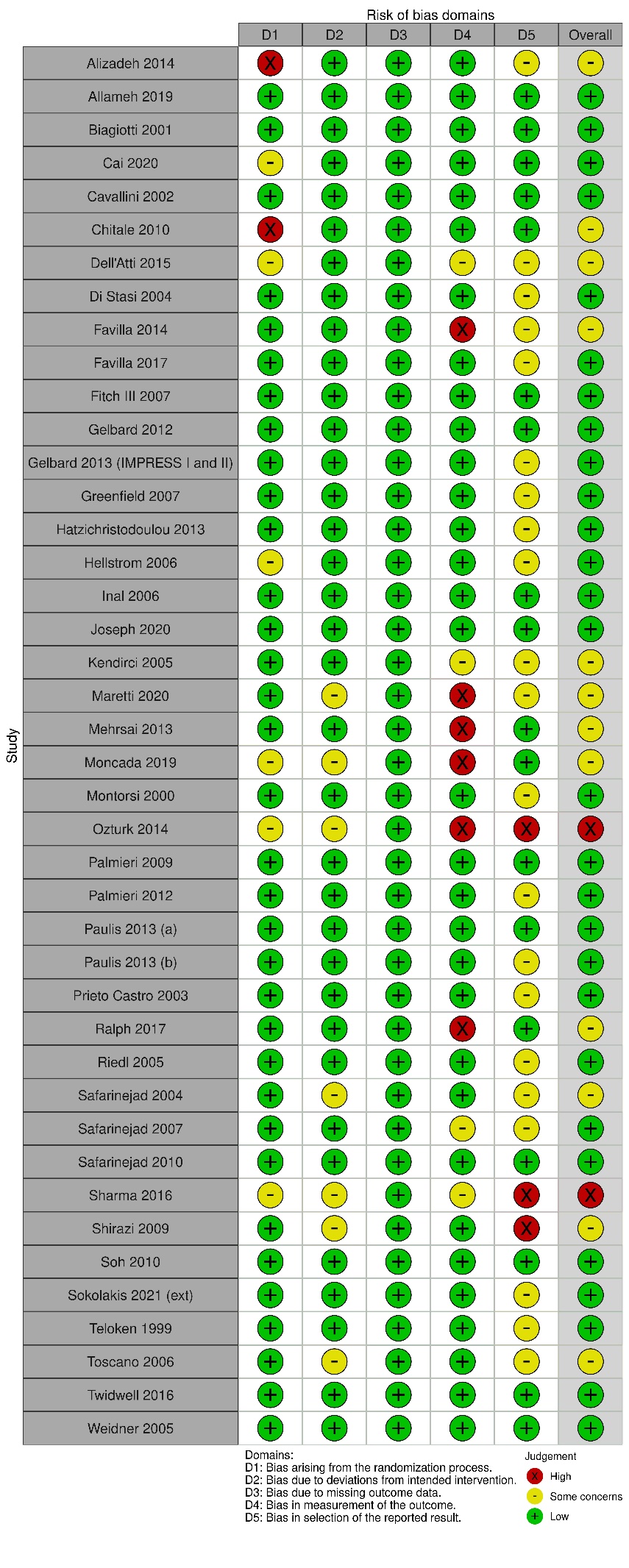
Appendix B- Risk of Bias Table

Appendix C-GRADE Evidence Tables

1.GRADE Evidence Table for Vitamin E

| **Certainty assessment** | | | | | | | **№ of patients** | | **Effect** | **Certainty** | **Importance** |
| --- | --- | --- | --- | --- | --- | --- | --- | --- | --- | --- | --- |
| **№ of studies** | **Study design** | **Risk of bias** | **Inconsistency** | **Indirectness** | **Imprecision** | **Other considerations** | **vitamin E** | **[control]** |  |  |  |
| **Penile curvature** | | | | | | | | | | | |
| 4 | randomised trials | not serious | serious | not serious | not serious | none | 148 | 148 | -12.25 to -8.7 (range for two studies)  Improvement of 48% in one study | ⨁⨁⨁◯ MODERATE | CRITICAL |
| **Plaque size** | | | | | | | | | | | |
| 4 | randomised trials | not serious | serious | not serious | not serious | none | 148 | 148 | Improvement range of 0.26cm-5.02cm in two studies and 30.8% reduction in one study | ⨁⨁⨁◯ MODERATE | IMPORTANT |
| **Pain** | | | | | | | | | | | |
| 4 | randomised trials | not serious | serious | not serious | not serious | none | 148 | 148 | No evidence for improvement | ⨁⨁⨁◯ MODERATE | CRITICAL |
| **IIEF** | | | | | | | | | | | |
| 3 | randomised trials | not serious | serious | not serious | not serious | none | 125 | 126 | Improvement range of 4.9 to 5.07 in two studies | ⨁⨁⨁◯ MODERATE | IMPORTANT |
| **Penile length** | | | | | | | | | | | |
| Nil | N/A | N/A | N/A | N/A | N/A | N/A | N/A | N/A | N/A | N/A | N/A |

2.GRADE Evidence Table for Tamoxifen

| **Certainty assessment** | | | | | | | **№ of patients** | | **Effect** | | **Certainty** | **Importance** |
| --- | --- | --- | --- | --- | --- | --- | --- | --- | --- | --- | --- | --- |
| **№ of studies** | **Study design** | **Risk of bias** | **Inconsistency** | **Indirectness** | **Imprecision** | **Other considerations** | **tamoxifen[intervention]** | **[comparison]** |  |  |  |  |
| **Penile curvature** | | | | | | | | | | | | |
| 1 | randomised trials | serious | serious | not serious | serious | none | 13 | 12 | No consistent evidence for improvement | | ⨁◯◯◯ VERY LOW | CRITICAL |
| Plaque size | | | | | | | | | | | | |
| 1 | randomised trials | serious | not serious | not serious | serious | none | 13 | 12 | No evidence for improvement | | ⨁⨁◯◯ LOW | IMPORTANT |
| Pain | | | | | | | | | | | | |
| 1 | randomised trials | serious | serious | not serious | serious | none | 13 | 12 | No consistent evidence for improvement | | ⨁◯◯◯ VERY LOW | CRITICAL |
| **IIEF** | | | | | | | | | | | | |
| Nil | N/A | N/A | N/A | N/A | N/A | N/A | N/A | N/A | N/A | | N/A | IMPORTANT |
| **Penile length** | | | | | | | | | | | | |
| Nil | N/A | N/A | N/A | N/A | N/A | N/A | N/A | N/A | N/A | | -N/A | IMPORTANT |

3.GRADE Evidence Table for POTABA

| **Certainty assessment** | | | | | | | **№ of patients** | | **Effect** | **Certainty** | **Importance** |
| --- | --- | --- | --- | --- | --- | --- | --- | --- | --- | --- | --- |
| **№ of studies** | **Study design** | **Risk of bias** | **Inconsistency** | **Indirectness** | **Imprecision** | **Other considerations** | **POTABA [intervention]** | **[comparison]** |  |  |  |
| **penile curvature** | | | | | | | | | | | |
| 1 | randomised trials | serious | not serious | not serious | serious | none | 51 | 52 | 74.3% had reduction of at least 30% | ⨁⨁◯◯ LOW | CRITICAL |
| **plaque size** | | | | | | | | | | | |
| 1 | randomised trials | serious | not serious | not serious | serious | none | 51 | 52 | No absolute figure given however states significantly reduced | ⨁⨁◯◯ LOW | IMPORTANT |
| **pain** | | | | | | | | | | | |
| 1 | randomised trials | serious | serious | not serious | serious | none | 51 | 52 | No evidence for significant improvement | ⨁◯◯◯ VERY LOW | CRITICAL |
| **IIEF** | | | | | | | | | | | |
| **1** | randomised trials | serious | serious | not serious | serious | none | 51 | 52 | No evidence for significant improvement | ⨁◯◯◯ VERY LOW | IMPORTANT |
| **PENILE length** | | | | | | | | | | | |
| **0** | - | - | - | - | - | - | - | - | - | - | IMPORTANT |

4.GRADE Evidence Table for PDEi-5

| **Certainty assessment** | | | | | | | **№ of patients** | | **Effect** | **Certainty** | **Importance** |
| --- | --- | --- | --- | --- | --- | --- | --- | --- | --- | --- | --- |
| **№ of studies** | **Study design** | **Risk of bias** | **Inconsistency** | **Indirectness** | **Imprecision** | **Other considerations** | **PDEi-5[intervention]** | **[comparison]** |  |  |  |
| **Penile curvature** | | | | | | | | | | | |
| 1 | randomised trials | serious | not serious | not serious | serious | none | 21 | 19 | No evidence for improvement | ⨁⨁◯◯ LOW | CRITICAL |
| **plaque size** | | | | | | | | | | | |
| 1 | randomised trials | serious | serious | not serious | serious | none | 21 | 19 | No evidence for improvement | ⨁◯◯◯ VERY LOW | IMPORTANT |
| **pain** | | | | | | | | | | | |
| 1 | randomised trials | serious | not serious | not serious | serious | none | 21 | 19 | Reduction of pain score of 1.29 | ⨁⨁◯◯ LOW | CRITICAL |
| **IIEF** | | | | | | | | | | | |
| 1 | randomised trials | serious | not serious | not serious | serious | none | 21 | 19 | increase of 3.8 vs. 0.89 | ⨁⨁◯◯ LOW | IMPORTANT |
| **PENILE length** | | | | | | | | | | | |
| **0** | - | - | - | - | - | - | - | - | - | - | IMPORTANT |

5.GRADE Evidence Table for Colchicine

| Certainty assessment | | | | | | | № of patients | | Effect | Certainty | Importance |
| --- | --- | --- | --- | --- | --- | --- | --- | --- | --- | --- | --- |
| № of studies | Study design | Risk of bias | Inconsistency | Indirectness | Imprecision | Other considerations | Colchicine[intervention] | [comparison] |  |  |  |
| penile curvature | | | | | | | | | | | |
| 1 | randomised trials | serious | not serious | not serious | serious | none | 42 | 42 | NO Evidence for significant improvement | ⨁⨁◯◯ LOW | CRITICAL |
| plaque size | | | | | | | | | | | |
| 1 | randomised trials | serious | serious | not serious | serious | none | 42 | 42 | NO Evidence for significant improvement | ⨁◯◯◯ VERY LOW | IMPORTANT |
| pain | | | | | | | | | | | |
| 1 | randomised trials | serious | serious | not serious | serious | none | 42 | 42 | NO Evidence for significant improvement | ⨁◯◯◯ VERY LOW | CRITICAL |
| IIEF | | | | | | | | | | | |
| 0 | - | - | - | - | - | - | - | - | - | - | IMPORTANT |
| PENILE LENGTH | | | | | | | | | | | |
| 0 | - | - | - | - | - | - | - | - | - | - | IMPORTANT |

6.GRADE Evidence Table for Carnitine

| **Certainty assessment** | | | | | | | **№ of patients** | | **Effect** | **Certainty** | **Importance** |
| --- | --- | --- | --- | --- | --- | --- | --- | --- | --- | --- | --- |
| **№ of studies** | **Study design** | **Risk of bias** | **Inconsistency** | **Indirectness** | **Imprecision** | **Other considerations** | **carnitine[intervention]** | **[comparison]** |  |  |  |
| **penile curvature** | | | | | | | | | | | |
| 2 | randomised trials | serious | serious | not serious | serious | none | 83 | 83 | Improvement of 7.5 degrees in one study | ⨁◯◯◯ VERY LOW | CRITICAL |
| **plaque size** | | | | | | | | | | | |
| 2 | randomised trials | serious | serious | not serious | serious | none | 83 | 83 | Improvement of 26.9mm in one study | ⨁◯◯◯ VERY LOW | IMPORTANT |
| **pain** | | | | | | | | | | | |
| 2 | randomised trials | serious | serious | not serious | serious | none | 83 | 83 | No evidence for significant improvement | ⨁◯◯◯ VERY LOW | CRITICAL |
| **IIEF** | | | | | | | | | | | |
| 1 | randomised trials | serious | not serious | not serious | serious | none | 59 | 59 | No evidence for significant improvement | ⨁⨁◯◯ LOW | IMPORTANT |
| **PENILE length** | | | | | | | | | | | |
| **0** | - | - | - | - | - | - | - | - | - | - | IMPORTANT |

7.GRADE Evidence Table for Co-Enzyme Q10

| **Certainty assessment** | | | | | | | **№ of patients** | | **Effect** | **Certainty** | **Importance** |
| --- | --- | --- | --- | --- | --- | --- | --- | --- | --- | --- | --- |
| **№ of studies** | **Study design** | **Risk of bias** | **Inconsistency** | **Indirectness** | **Imprecision** | **Other considerations** | **co-enzymeQ10[intervention]** | **[comparison]** |  |  |  |
| **penile curvature** | | | | | | | | | | | |
| 1 | randomised trials | serious | not serious | not serious | serious | none | 93 | 93 | 60.5% have improvement in one study | ⨁⨁◯◯ LOW | CRITICAL |
| **plaque size** | | | | | | | | | | | |
| 1 | randomised trials | serious | not serious | not serious | serious | none | 93 | 93 | 43% improvement in one study | ⨁⨁◯◯ LOW | IMPORTANT |
| **pain** | | | | | | | | | | | |
| 1 | randomised trials | serious | not serious | not serious | serious | none | 93 | 93 | No evidence for significant improvement | ⨁⨁◯◯ LOW | CRITICAL |
| **IIEF** | | | | | | | | | | | |
| 1 | randomised trials | serious | not serious | not serious | serious | none | 93 | 93 | Improvement of 9.6 | ⨁⨁◯◯ LOW | IMPORTANT |
| **penile length** | | | | | | | | | | | |
| **0** | - | - | - | - | - | - | - | - | - | - | IMPORTANT |

8.GRADE Evidence Table for intralesional CCH

| **Certainty assessment** | | | | | | | **№ of patients** | | **Effect** | **Certainty** | **Importance** |
| --- | --- | --- | --- | --- | --- | --- | --- | --- | --- | --- | --- |
| **№ of studies** | **Study design** | **Risk of bias** | **Inconsistency** | **Indirectness** | **Imprecision** | **Other considerations** | **intralesional CCH** | **[comparison]** |  |  |  |
| **Penile curvature** | | | | | | | | | | | |
| 2 | randomised trials | not serious | not serious | not serious | serious | strong association | 512 | 247 | Improvement range 16.3-17 degrees | ⨁⨁⨁◯ MODERATE | CRITICAL |
| **plaque size** | | | | | | | | | | | |
| 0 | - | - | - | - | - | - | - | - | - | - | IMPORTANT |
| **pain** | | | | | | | | | | | |
| 1 | randomised trials | not serious | not serious | not serious | serious | none | 401 | 211 | No significant improvement in pain | ⨁⨁⨁◯ MODERATE | CRITICAL |
| **IIEF** | | | | | | | | | | | |
| 2 | randomised trials | not serious | serious | not serious | serious | none | 512 | 247 | Significant Improvement of IIEF score of 1.0 in one study | ⨁⨁◯◯ LOW | IMPORTANT |
| **penile length** | | | | | | | | | | | |
| 2 | randomised trials | not serious | serious | not serious | serious | none | 512 | 247 | Improvement of 0.4cm in one study | ⨁⨁◯◯ LOW | IMPORTANT |

9. GRADE Evidence Table for interferon

| **Certainty assessment** | | | | | | | **№ of patients** | | **Effect** | **Certainty** | **Importance** |
| --- | --- | --- | --- | --- | --- | --- | --- | --- | --- | --- | --- |
| **№ of studies** | **Study design** | **Risk of bias** | **Inconsistency** | **Indirectness** | **Imprecision** | **Other considerations** | **interferon[intervention]** | **[comparison]** |  |  |  |
| **penile curvature** | | | | | | | | | | | |
| 2 | randomised trials | serious | serious | not serious | serious | none | 69 | 73 | Improvement of 12.0-13.5 degrees | ⨁◯◯◯ VERY LOW | CRITICAL |
| **plaque size** | | | | | | | | | | | |
| 2 | randomised trials | serious | not serious | not serious | serious | none | 69 | 73 | Improvement of 2.2-1.67 cm^2^ | ⨁⨁◯◯ LOW | IMPORTANT |
| **pain** | | | | | | | | | | | |
| 2 | randomised trials | serious | not serious | not serious | serious | none | 69 | 73 | Improved in 58-67% | ⨁⨁◯◯ LOW | CRITICAL |
| **IIEF** | | | | | | | | | | | |
| 2 | randomised trials | serious | not serious | not serious | serious | none | 69 | 73 | No evidence for significant improvement | ⨁⨁◯◯ LOW | IMPORTANT |
| **penile length** | | | | | | | | | | | |
| **0** | - | - | - | - | - | - | - | - | - | - | IMPORTANT |

10.GRADE Evidence Table for intralesional CCB

| **Certainty assessment** | | | | | | | **№ of patients** | | **Effect** | | **Certainty** | **Importance** |
| --- | --- | --- | --- | --- | --- | --- | --- | --- | --- | --- | --- | --- |
| **№ of studies** | **Study design** | **Risk of bias** | **Inconsistency** | **Indirectness** | **Imprecision** | **Other considerations** | **intralesional CCB[intervention]** | **[comparison]** | **Relative (95% CI)** | **Absolute (95% CI)** |  |  |
| **penile curvature** | | | | | | | | | | | | |
| 4 | randomised trials | not serious | very serious | not serious | not serious | none | 152 | 139 | No evidence of improvement | | ⨁⨁◯◯ LOW | CRITICAL |
| **plaque size** | | | | | | | | | | | | |
| 4 | randomised trials | not serious | very serious | not serious | not serious | none | 152 | 139 | Improvement of 12 mm in one study | | ⨁⨁◯◯ LOW | IMPORTANT |
| **pain** | | | | | | | | | | | | |
| 4 | randomised trials | not serious | very serious | not serious | not serious | none | 152 | 139 | 76.7% had improvement in pain in one study | | ⨁⨁◯◯ LOW | CRITICAL |
| **IIEF** | | | | | | | | | | | | |
| 1 | Randomised trials | Not serious | Very serious | Not serious | serious | None | 37 | 37 | Significant improvement in one study | | ⨁◯◯◯ VERY LOW | IMPORTANT |
| **penile length** | | | | | | | | | | | | |
| 0 | - | - | - | - | - | - | - | - | - | | - | IMPORTANT |

11.GRADE Evidence Table for HA

| **Certainty assessment** | | | | | | | **№ of patients** | | **Effect** | **Certainty** | **Importance** |
| --- | --- | --- | --- | --- | --- | --- | --- | --- | --- | --- | --- |
| **№ of studies** | **Study design** | **Risk of bias** | **Inconsistency** | **Indirectness** | **Imprecision** | **Other considerations** | **HA[intervention]** | **[comparison]** |  |  |  |
| **penile curvature** | | | | | | | | | | | |
| 1 | randomised trials | serious | not serious | not serious | serious | none | 63 | 69 | -  Improvement of 4.6 degrees in one study | ⨁⨁◯◯ LOW | CRITICAL |
| **plaque size** | | | | | | | | | | | |
| 1 | randomised trials | serious | serious | not serious | serious | none | 63 | 69 | -  No evidence of significant improvement | ⨁◯◯◯ VERY LOW | IMPORTANT |
| **pain** | | | | | | | | | | | |
| 0 |  |  |  |  |  |  |  |  | - |  | CRITICAL |
| **IIEF** | | | | | | | | | | | |
| 1 | randomised trials | serious | serious | not serious | serious | none | 63 | 69 | -  **No evidence of significant improvement** | ⨁◯◯◯ VERY LOW | IMPORTANT |
| **PENILE length** | | | | | | | | | | | |
| 0 | - | - | - | - | - | - | - | - | - | - | IMPORTANT |

12.GRADE Evidence Table for intralesional thiocolchicine

| **Certainty assessment** | | | | | | | **№ of patients** | | **Effect** | **Certainty** | **Importance** |
| --- | --- | --- | --- | --- | --- | --- | --- | --- | --- | --- | --- |
| **№ of studies** | **Study design** | **Risk of bias** | **Inconsistency** | **Indirectness** | **Imprecision** | **Other considerations** | **intralesional thiocolchicine[intervention]** | **[comparison]** |  |  |  |
| **penile curvature** | | | | | | | | | | | |
| 1 | randomised trials | serious | not serious | not serious | serious | none | 13 | 12 | **Improvement of 10.54 degrees in one study** | ⨁⨁◯◯ LOW | CRITICAL |
| **plaque size** | | | | | | | | | | | |
| 1 | randomised trials | serious | not serious | not serious | serious | none | 13 | 12 | -  **No evidence for significant improvement** | ⨁⨁◯◯ LOW | IMPORTANT |
| **pain** | | | | | | | | | | | |
| 0 | - | - | - | - | - | - | - | - | - | - | CRITICAL |
| **IIEF** | | | | | | | | | | | |
| 1 | randomised trials | serious | not serious | not serious | serious | none | 13 | 12 | **No evidence for significant improvement** | ⨁⨁◯◯ LOW | IMPORTANT |
| **PENILE Length** | | | | | | | | | | | |
| **0** | - | - | - | - | - | - | - | - | - | - | IMPORTANT |

13.GRADE Evidence Table for ESWT

| **Certainty assessment** | | | | | | | **№ of patients** | | **Effect** | **Certainty** | **Importance** |
| --- | --- | --- | --- | --- | --- | --- | --- | --- | --- | --- | --- |
| **№ of studies** | **Study design** | **Risk of bias** | **Inconsistency** | **Indirectness** | **Imprecision** | **Other considerations** | **ESWT [intervention]** | **[comparison]** |  |  |  |
| **penile curvature** | | | | | | | | | | | |
| 3 | randomised trials | serious | not serious | not serious | serious | none | 117 | 121 | Improvement of 1.43 degrees in one study | ⨁⨁◯◯ LOW | CRITICAL |
| **plaque size** | | | | | | | | | | | |
| 2 | randomised trials | not serious | serious | not serious | not serious | none | 101 | 101 | Improvement of 0.06cm2 in one study | ⨁⨁⨁◯ MODERATE | IMPORTANT |
| **pain** | | | | | | | | | | | |
| 3 | randomised trials | serious | not serious | not serious | serious | none | 117 | 121 | Improvement of 2.5-5.05 in two studies | ⨁⨁◯◯ LOW | CRITICAL |
| **IIEF** | | | | | | | | | | | |
| 2 | randomised trials | not serious | serious | not serious | not serious | none | 66 | 70 | Improvement of 5.4 in one study | ⨁⨁⨁◯ MODERATE | IMPORTANT |
| **PENILE length** | | | | | | | | | | | |
| 1 | randomised trials | not serious | serious | not serious | serious | none | 16 | 20 | No evidence for significant improvement | ⨁⨁◯◯ LOW | IMPORTANT |

14.GRADE Evidence Table for Electromotive therapy

| Certainty assessment | | | | | | | № of patients | | Effect | Certainty | Importance |
| --- | --- | --- | --- | --- | --- | --- | --- | --- | --- | --- | --- |
| № of studies | Study design | Risk of bias | Inconsistency | Indirectness | Imprecision | Other considerations | electromotive[intervention] | [comparison] |  |  |  |
| penile curvature | | | | | | | | | | | |
| 4 | randomised trials | not serious | serious | not serious | not serious | none | 110 | 108 | Improvement of 22 degrees in one study. 62% had improvement of penile length in another study | ⨁⨁⨁◯ MODERATE | CRITICAL |
| plaque size | | | | | | | | | | | |
| 3 | randomised trials | not serious | serious | not serious | not serious | none | 87 | 86 | Improvement in plaque volume of 477.4mm3 in one study. In another study 79% had improvement | ⨁⨁⨁◯ MODERATE | IMPORTANT |
| pain | | | | | | | | | | | |
| 3 | randomised trials | not serious | serious | not serious | not serious | none | 87 | 86 | Reduction of 4.1 in pain score in one study | ⨁⨁⨁◯ MODERATE | CRITICAL |
| IIEF | | | | | | | | | | | |
| 1 | randomised trials | not serious | serious | not serious | not serious | none | 30 | 30 | No evidence of significant improvement | ⨁⨁◯◯ LOW | IMPORTANT |
| PENILE length | | | | | | | | | | | |
| 1 | randomised trials | not serious | not serious | not serious | serious | none | 30 | 30 | 76.6% experienced shortening (in those that responded to treatment) | ⨁⨁◯◯ LOW | IMPORTANT |

15.GRADE Evidence Table for Traction devices

| Certainty assessment | | | | | | | № of patients | | Effect | Certainty | Importance |
| --- | --- | --- | --- | --- | --- | --- | --- | --- | --- | --- | --- |
| № of studies | Study design | Risk of bias | Inconsistency | Indirectness | Imprecision | Other considerations | traction device [intervention] | [comparison] |  |  |  |
| penile curvature | | | | | | | | | | | |
| 2 | randomised trials | not serious | not serious | not serious | serious | none | 70 | 66 | Improvement ranged from -38.4 to -8.5 degrees | ⨁⨁⨁◯ MODERATE | CRITICAL |
| plaque size | | | | | | | | | | | |
| 0 | - | - | - | - | - | - | - | - | - | - | IMPORTANT |
| pain | | | | | | | | | | | |
| 1 | randomised trials | not serious | not serious | not serious | serious | none | 29 | 27 | No significant evidence for improvement | ⨁⨁⨁◯ MODERATE | CRITICAL |
| IIEF | | | | | | | | | | | |
| 2 | randomised trials | not serious | not serious | not serious | serious | none | 70 | 66 | No significant evidence for improvement | ⨁⨁⨁◯ MODERATE | IMPORTANT |
| penile length | | | | | | | | | | | |
| 2 | randomised trials | not serious | not serious | not serious | serious | none | 70 | 66 | Improvement of 1.5 cm in one study | ⨁⨁⨁◯ MODERATE | IMPORTANT |

16.GRADE Evidence Table for Laser

| Certainty assessment | | | | | | | № of patients | | Effect | | Certainty | Importance |
| --- | --- | --- | --- | --- | --- | --- | --- | --- | --- | --- | --- | --- |
| № of studies | Study design | Risk of bias | Inconsistency | Indirectness | Imprecision | Other considerations | laser[intervention] | [comparison] | Relative (95% CI) | Absolute (95% CI) |  |  |
| penile curvature | | | | | | | | | | | | |
| 1 | randomised trials | not serious | not serious | not serious | serious | none | 18 | 20 | Improvement of 5 degrees in one study | | ⨁⨁⨁◯ MODERATE | CRITICAL |
| plaque size | | | | | | | | | | | | |
| 1 | randomised trials | not serious | not serious | not serious | serious | none | 18 | 20 | No evidence for improvement | | ⨁⨁⨁◯ MODERATE | IMPORTANT |
| pain | | | | | | | | | | | | |
| 1 | randomised trials | not serious | not serious | not serious | serious | none | 18 | 20 | Improvement of 2.65 in VAS pain score in one study | | ⨁⨁⨁◯ MODERATE | CRITICAL |
| IIEF | | | | | | | | | | | | |
| 1 | randomised trials | not serious | not serious | not serious | serious | none | 18 | 20 | Improvement of 7.05 in one study | | ⨁⨁⨁◯ MODERATE | IMPORTANT |
| penile length | | | | | | | | | | | | |
| 1 | randomised trials | not serious | not serious | not serious | serious | none | 18 | 20 | - | | ⨁⨁⨁◯ MODERATE | IMPORTANT |

17.GRADE Evidence Table for Topical Therapy

| **Certainty assessment** | | | | | | | **№ of patients** | | **Effect** | **Certainty** | **Importance** |
| --- | --- | --- | --- | --- | --- | --- | --- | --- | --- | --- | --- |
| **№ of studies** | **Study design** | **Risk of bias** | **Inconsistency** | **Indirectness** | **Imprecision** | **Other considerations** | **Topical Therapy[intervention]** | **[comparison]** |  |  |  |
| **penile curvature** | | | | | | | | | | | |
| 3 | randomised trials | serious | serious | not serious | not serious | none | 59 | 59 | One study demonstrated subjective improvements | ⨁⨁◯◯ LOW | CRITICAL |
| **plaque size** | | | | | | | | | | | |
| 2 | randomised trials | not serious | serious | not serious | serious | none | 48 | 48 | 55-84.7% had change in plaque size in two studies | ⨁⨁◯◯ LOW | IMPORTANT |
| **pain** | | | | | | | | | | | |
| 3 | randomised trials | not serious | serious | not serious | serious | none | 59 | 59 | Reduction of pain score of 2.1 in one study, in two studies 88-100% had reduction in pain | ⨁⨁◯◯ LOW | CRITICAL |
| **IIEF** | | | | | | | | | | | |
| 0 | - | - | - | - | - | - | - | - | - | - | IMPORTANT |
| **penile length** | | | | | | | | | | | |
| 1 | randomised trials | not serious | serious | not serious | serious | none | 11 | 11 | Improvement of 2.2.cm in one study | ⨁⨁◯◯ LOW | IMPORTANT |
